# SOCCOMAS: a FAIR web content management system that uses knowledge graphs and that is based on semantic programming

**DOI:** 10.1093/database/baz067

**Published:** 2019-08-08

**Authors:** Lars Vogt, Roman Baum, Philipp Bhatty, Christian Köhler, Sandra Meid, Björn Quast, Peter Grobe

**Affiliations:** 1 Institut für Evolutionsbiologie und Ökologie, Rheinische Friedrich-Wilhelms-Universität Bonn, An der Immenburg 1, 53121 Bonn, Germany; 2 Zoologisches Forschungsmuseum Alexander Koenig, Adenauerallee 160, 53113 Bonn, Germany

## Abstract

We introduce Semantic Ontology-Controlled application for web Content Management Systems (SOCCOMAS), a development framework for FAIR (‘findable’, ‘accessible’, ‘interoperable’, ‘reusable’) Semantic Web Content Management Systems (S-WCMSs). Each S-WCMS run by SOCCOMAS has its contents managed through a corresponding knowledge base that stores all data and metadata in the form of semantic knowledge graphs in a Jena tuple store. Automated procedures track provenance, user contributions and detailed change history. Each S-WCMS is accessible via both a graphical user interface (GUI), utilizing the JavaScript framework AngularJS, and a SPARQL endpoint. As a consequence, all data and metadata are maximally findable, accessible, interoperable and reusable and comply with the FAIR Guiding Principles. The source code of SOCCOMAS is written using the Semantic Programming Ontology (SPrO). SPrO consists of commands, attributes and variables, with which one can describe an S-WCMS. We used SPrO to describe all the features and workflows typically required by any S-WCMS and documented these descriptions in a SOCCOMAS source code ontology (SC-Basic). SC-Basic specifies a set of default features, such as provenance tracking and publication life cycle with versioning, which will be available in all S-WCMS run by SOCCOMAS. All features and workflows specific to a particular S-WCMS, however, must be described within an instance source code ontology (INST-SCO), defining, e.g. the function and composition of the GUI, with all its user interactions, the underlying data schemes and representations and all its workflow processes. The combination of descriptions in SC-Basic and a given INST-SCO specify the behavior of an S-WCMS. SOCCOMAS controls this S-WCMS through the Java-based middleware that accompanies SPrO, which functions as an interpreter. Because of the ontology-controlled design, SOCCOMAS allows easy customization with a minimum of technical programming background required, thereby seamlessly integrating conventional web page technologies with semantic web technologies. SOCCOMAS and the Java Interpreter are available from (https://github.com/SemanticProgramming).

## Introduction

Every day we create more and more data, in exponentially increasing amounts. More than 90% of today’s data have been created within the past 2 years (1–3). With the emergence of high-throughput technologies, social media, mobile services, digital photos and the Internet of things, big data created in science and everyday life allow us to answer questions that could not be answered before, resulting in the advent of a new driving force for scientific progress that is becoming increasingly important in all data-rich fields of empirical research. This new approach to research has been called data exploration or eScience (4).

Big data and eScience are virtue and challenge at the same time. Challenges arise from the amounts of data, the rates in which they are created and transmitted, and from their heterogeneity. This change in size, velocity and variety that big data bring about outclasses the capabilities of conventional methods and techniques of handling, processing, analyzing, managing, storing and retrieving data within a reasonable time frame (5). In order to find the data that are relevant to a given study from within a lake of data and process them in a timely manner, we now have to rely on algorithms and software applications. eScience thus requires the development of corresponding applications and services that focus on capturing, curating, mining, integrating, analyzing and reasoning over data, thereby often utilizing Semantic Web technologies, web content management systems and data harvester services. The applications and services, in turn, demand data and accompanying metadata to be semantically structured in a standardized way that makes them computer-parsable and semantically transparent. Ontologies and other controlled vocabularies have taken a central role in this context (6–9).

More and more organizations and institutions recognize their research data to be their most valuable asset and seek for technical solutions for managing the accessibility, usability, disseminability, integrity and security of all the data they create. Content management systems, coupled with ontologies and semantic technology, have the potential to provide a solution that meets these new requirements from organizations and institutions as well as from eScience. Unfortunately, not many content management systems have implemented ontologies and semantic technology to their full potential. Our impression is that the overwhelming majority of applications of ontologies in the life sciences has been restricted to semantically enriching documents and annotating database contents by using Uniform Resource Identifiers (URIs) of ontology classes as values in tables of relational databases. Most content management systems do not document and communicate their data in the form of instance-based Resource Description Framework (RDF) (10) triple statements and thus do not benefit from the detailed search functions and the reasoning capabilities of tuple stores, including consistency checks, data analyses, data integration and reusability of data in for example mobile apps (11).

We do not think that this is due to technological limitations and restrictions. Tuple stores are capable of handling large volumes of RDF triple statements representing data and metadata as well as underlying data schemes (i.e. ontologies (11–14)). ‘Tuple store’ is a general expression for a store that stores tuples. A tuple is a list of entities. Several common specific expressions exist: a triple store, also called RDF store, is a tuple store that stores triples, i.e. subject–predicate–object expressions. A quad store is a tuple store that stores quadruples, i.e. subject–predicate–object–named graph expressions. In a tuple store, data are organized through a layer of triple statements that specify a hierarchy of classes and subclasses with accompanying axioms, i.e. ontologies (see ‘universal statements’ further below). The actual data are triple statements about instances of respective classes (see ‘assertional statements’ further below). The function of ontologies in a tuple store is thus comparable to the data scheme in a relational database. Semantic technology facilitates detailed information retrieval from large sets of triple statements through SPARQL endpoints (15) and reasoning over them through semantic reasoners (16).

Although tuple stores and RDF-based data solutions are superior in many respects, they yet have to replace conventional relational databases such as MySQL or PostgreSQL in rank as the prime database technology for content management systems. One reason is the lack of application development frameworks with a native graph data structure that are well integrated with RDF and allow handling graph data and manipulating and displaying SPARQL results (17) [for initial attempts of integrating RDF with conventional technologies, but not specifically with content management systems, see e.g. (18–27)].

After introducing some background on ontologies, semantic knowledge graphs, knowledge bases and today’s demands on web content management systems, we introduce a new approach to application programming in which the source code is written in an ontology, the source code ontology, using terms from the Semantic Programming Ontology (SPrO). We call this approach ‘semantic programming’ (28). The source code is interpreted by an accompanying Java-based middleware that executes it and produces the respective application. We then introduce Semantic Ontology-Controlled application for web Content Management Systems (SOCCOMAS), a Semantic Web Content Management System (S-WCMS) that is based on semantic programming and used for creating and publishing data documents. All S-WCMSs based on SOCCOMAS provide data and metadata complying with the FAIR (‘Findable’, ‘Accessible’, ‘Interoperable’, ‘Reusable’) Guiding Principles (29).

## Ontologies, empirical data, knowledge bases and semantic knowledge graphs

The use of ontologies for semantically enriching documents and annotating database contents promises to provide not only semantic transparency and computer parsability but also a framework for applying data and metadata standards that improve the integration and interoperability of data, all of which is much needed in the age of eScience (30–32).

Ontologies are dictionaries that can be used for describing a certain reality. They consist of labeled classes with clear definitions that are created by experts through consensus and that are formulated in a highly formalized canonical syntax and standardized format, such as the Web Ontology Language (OWL) that can be serialized to RDF, with the goal to yield a lexical or taxonomic framework for knowledge representation (33). Each ontology class possesses its own URI, through which it can be identified and individually referenced. Ontologies contain expert-curated domain knowledge about specific kinds of entities together with their properties and relations in the form of classes defined through universal statements (34, 35). Ontologies in this sense do not include statements about particular entities, that is, about individuals.

Statements about particular entities are assertional statements. Description logic (DL) distinguishes TBox and ABox expressions. TBoxes contain assertions on classes, whereas ABoxes contain assertions on instances. Class axioms expressed in OWL are TBox expressions, whereas empirical data statements are ABox expressions. If assertional statements are grounded in empirical knowledge that is based on observation and experimentation, we refer to them as empirical data. Both ontologies and data can be documented in the form of sets of triple statements, following RDF’s syntax of ‘Subject’, ‘Predicate’ and ‘Object’. Any particular entity mentioned in a data statement should have its own URI for reference, and its class affiliation should be specified by referencing the URI of the respective ontology class that the particular entity instantiates. In this way, empirical data become semantically transparent because they reference the ontology class that each of their described particular entities instantiates and with it also the class’s definition (see 3 below).

A given URI can take the ‘Object’ position in one triple statement and the ‘Subject’ position in another triple statement. As a consequence, several triple statements can be connected to form a network of RDF/OWL-based triple statements, i.e. a semantic graph. Because both ontologies and empirical data can be documented in RDF/OWL, we distinguish class-based and instance-based semantic graphs, respectively [representing data as an instance-based instead of a class-based semantic graph has many advantages (36)]. Empirical data represented as an instance-based semantic graph is computer-parsable, and algorithms can reason over them, thus taking full advantage of the power of Semantic Web technologies.

Obviously, not every OWL file and not every semantic graph is an ontology—it is an ontology only if it limits itself to express universal statements about kinds of entities (35). A knowledge base, in contrast, consists of a set of ontology classes that are populated with empirical data (35). Ontologies, therefore, do not represent knowledge bases but are part of them and provide a means to structure them (37) [An ontology contains TBox expressions, whereas a knowledge base expressed in DL is constituted by a combination of TBox and ABox expressions (38).]. In this sense, one can think of ontologies as providing the data schemes in knowledge bases.

**Figure 1:**
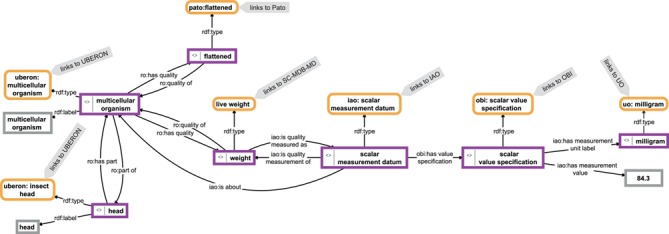
Example for a knowledge graph. The knowledge graph describes parts of the anatomy of a particular multicellular organism that is of a flattened shape, has a weight of 84.3 mg and has a head as its part. The description is not comprehensive and follows the open world assumption. Parts of the graph specify relations between instances (purple-bordered boxes) or specify values or labels referring to instances (gray-bordered boxes) and thus represent instance-based subgraphs, whereas other parts link to class expressions (yellow-bordered boxes), which in turn link to class-based subgraphs provided by the respective ontologies (IAO, information artifact ontology (44); OBI, ontology for biomedical investigations (45); PATO, phenotype quality ontology (46); SC-MDB-MD, semantic Morph·D·Base source code ontology for the morphological descriptions module; UBERON, Uber-anatomy ontology (47); UO, units of measurement ontology (48)). For reasons of clarity, resources are not represented with their URIs but with labels.

The contents of such a knowledge base form a semantic knowledge graph (39–43) (Figure 1). We use the term ‘semantic knowledge graph’ here to be understood as the combination of instance-based semantic graphs that contain assertional statements (i.e. data) and class-based semantic graphs that contain universal statements (i.e. ontologies). Semantic knowledge graphs are thus RDF-based knowledge graphs.

## eScience-compliant data and metadata standards and the FAIR Guiding Principles

To be maximally efficient, eScience requires the development and implementation of eScience-compliant data and metadata standards that establish semantic transparency and computer parsability. This is achieved by providing for each data or metadata statement the adequate nomenclatural, concept, format and content standards (9, 31, 32), which together also guarantees their FAIRness.

An eScience-compliant ‘nomenclatural standard’ unambiguously links the words and identifiers used in a data or metadata statement to their underlying concepts, whereas an eScience-compliant ‘concept standard’ specifies the meaning of these concepts. Many ontologies provide formalized term definitions accompanied by human-readable free text definitions that provide concept standards. Moreover, since each ontology term has its own persistent URI in addition to a human-readable label and some ontologies even specify additional human-readable synonyms, ontologies also provide the required nomenclatural standards. In a semantic knowledge graph, the URIs of the instantiated ontology classes guarantee that data and metadata are ‘findable’ and ‘interoperable’ across all knowledge bases that use the respective ontology classes as part of their data scheme. Moreover, data and metadata of a knowledge base are ‘accessible’ through the SPARQL endpoint of the knowledge base. If the URIs of the instantiated ontology classes are also URLs, they are ‘accessible’ through HTTP as well.

An eScience-compliant ‘content standard’ requires the specification of what information is relevant and must be covered for a particular type of data or metadata statement. This can be achieved by developing and implementing data models that specify for the scientific domain in question which information is relevant for a given type of data or metadata statement (9) [see also ‘minimum information convention’ (6, 7)]. Each such data model should take the form of a template graph for how to represent the respective type of data or metadata as a semantic knowledge graph [for a detailed discussion see (36, 49)]. In this way, a web content management system can implement procedures for automatically tracking provenance and user contributions to a given data document, thus contributing to a rich metadata documentation. This metadata should be documented as a semantic knowledge graph that can be searched through a SPARQL endpoint, which would further increase the ‘findability’ and ‘accessibility’ of the data. The content standard also ensures that data and metadata are richly described and meet domain-relevant community standards, which increases their ‘interoperability’ and ‘reusability’.

Last but not least, an eScience-compliant ‘format standard’ specifies the syntax and file format in which data or metadata statements should be recorded, archived and communicated in the Web. Here, OWL, RDF and RDF Schema (RDFS), which can be represented in Extensible Markup Language (XML) format or JavaScript Object Notation (JSON) (50), seem to become common consensus formats, although alternatives exist. They provide formal, accessible, shared and broadly applicable languages for knowledge representation and thus make data and metadata of respective semantic knowledge graphs ‘interoperable’.

If data and metadata statements comply with these four standards, they thus become maximally ‘findable’, ‘accessible’, ‘interoperable’ and ‘reusable’ and therefore comply with the FAIR Guiding Principles (29). In order to be FAIR, however, web content management systems must store and organize not only the scientific data in the form of semantic knowledge graphs but also their provenance and all other types of associated metadata, including change history, versioning and access rights (40, 51–54).

## S-WCMSs and their specific challenges

By S-WCMS we understand a web content management system that is based on a knowledge base, i.e. a set of ontology classes that are populated with empirical data. An S-WCMS is used for creating and publishing data documents. It stores its data and metadata in the form of eScience-compliant semantic knowledge graphs that provide FAIR data and metadata. A tuple store provides an efficient means to store and query semantic knowledge graphs. The data and metadata graphs can be readily consumed by various applications through the SPARQL endpoint of the S-WCMS.

The downside of storing data and metadata as semantic knowledge graphs, however, is that they often possess a rather complicated structure, which is why they are usually not as intuitively comprehensible for a human reader as data and metadata represented in form of conventional tables or entry forms. As a consequence, most human readers are not interested in directly interacting with semantic knowledge graphs. Unfortunately, SPARQL endpoints only allow interacting directly with semantic knowledge graphs and do not provide user-friendly presentations of data such as through HTML pages. Tools such as YASGUI (55) and Wikidata Query Service (56) do help to make SPARQL more accessible, but there is still a long way to go. In order to be intuitively accessible, S-WCMSs would have to hide all semantic knowledge graphs from their users and, instead, provide more user-friendly representations of their data and metadata. However, they would still have to store and manage their data and metadata in the form of semantic knowledge graphs in order to benefit from the advantages of ontologies and semantic technologies such as providing FAIR data and metadata and being capable of reasoning. What is required in order to increase the applicability of semantic graphs is a means for readers to indirectly interact with them through entry forms, tables and other ways of visualizing and interacting with data in intuitive ways.

Independent of the requirements for eScience, for FAIR data and metadata and for more intuitive ways of interacting with semantic graphs through graphical user interfaces (GUIs), we know from our own experience with running the morphological data repository Morph·D·Base (57), which has been online since 2006, that there is also a strong demand for a flexible middleware/backend that is easily adaptable to changing standards, project-specific requirements and newly emerging scientific workflows. After the initial funding phase of Morph·D·Base, we frequently faced the situation that users asked for extending given entry forms to allow for documenting additional data or metadata that were relevant for their projects and that we had not anticipated as relevant when we developed Morph·D·Base. Since Morph·D·Base is based on the relational database management system MySQL (MySQL 5.5; http://www.mysql.com) with a Zope middleware (http://www.zope.org), we usually had to program in three layers, (i) backend, (ii) middleware and (iii) frontend, using three different programming frameworks, which was tedious, error-prone and time consuming. We thus learned the hard way that, for long-term acceptance and overall longevity and sustainability of a scientific S-WCMS, it is crucial to be able to adapt it to individual needs and evolving demands without requiring substantial additional programming in three different layers. We thus wanted to develop a highly configurable system that is, ideally, both easy to use and to maintain and whose flexibility goes beyond being able to conveniently modify and adapt the GUI. Web content management systems that are based on conventional relational database technologies fail to provide such flexibility.

## SOCCOMAS

### Initial idea for SOCCOMAS

SOCCOMAS is an S-WCMS that is used for creating and publishing data documents that comply with the FAIR data principles. It is based on semantic programming and stores all data and metadata in the form of semantic knowledge graphs.

Instead of limiting the application of ontologies to modeling a specific domain, we had the idea to use ontologies also for software development. We developed SPrO with an accompanying Java-based middleware and interface that can be used like a programming language (28), with which we can describe and thereby control every aspect of a domain-independent S-WCMS. The descriptions themselves are formulated in OWL and stored in a corresponding ontology. The descriptions function like the source code of the S-WCMS, which is why we refer to the ontology as ‘source code ontology’. We realized that, by understanding the definition of the underlying data model and the specification of the S-WCMS as being equivalent to programming code, we would be able to implement new features and new types of data documents for any particular S-WCMS by describing them in a set of source code ontologies using SPrO. The source code ontologies thus provide the steering logic for an S-WCMS run by SOCCOMAS. With this clear separation of steering logic from interpretation logic, semantic programming follows the idea of separating main layers of an application, analogous to the separation of interpretation logic and presentation logic.

In other words, the underlying key idea of SOCCOMAS is that specific ontology resources in the form of classes, individuals and properties are defined in SPrO and can be used in a source code ontology for describing a data-centric application. The middleware interprets these SPrO classes, individuals and properties as commands, subcommands and variables [see (28)]. With SPrO and its accompanying middleware, semantic programming provides a basic development framework that supports developers of knowledge graph applications. The middleware provides a RESTful API that enables CRUD (i.e. create, read/retrieve, update and delete) operations on RDF data to be handled more easily for HTML/JavaScript developers. The API provides graph data within a JSON object together with structured information (just like a Document Object Model tree) for its HTML representation, which frontend developers can use to visualize a web page with data from the tuple store. It thus enables RDF data to be reused across languages, implementations, and libraries.

**Figure 2:**
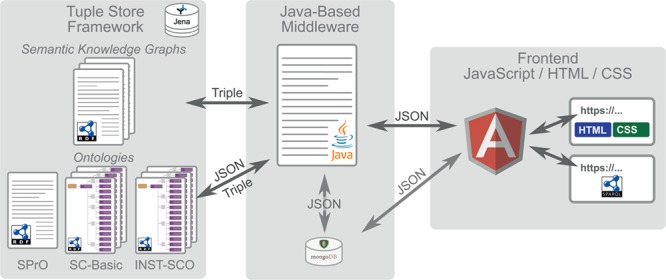
Overall workflow of SOCCOMAS. Left: Jena tuple store framework containing the data of the S-WCMS as well as a set of ontologies. The ontologies comprise (i) the SPrO (28), which defines the commands, subcommands and variables used for describing an S-WCMS, (ii) the SOCCOMAS SC-Basic, which contains the descriptions of general workflows and features that can be used by any S-WCMSO and (iii) an instance source code ontology (INST-SCO) for a particular S-WCMS, which has been individually customized to contain the descriptions of all features that are special to this particular S-WCMS. The data of the S-WCMS are stored in form of semantic knowledge graphs. Middle: The Java-based middleware with associated MongoDB reads the descriptions contained in SC-Basic and INST-SCO and interprets them as the specification of this particular S-WCMS. Right: The frontend, based on the JavaScript framework AngularJS, with HTML and CSS output for browser requests and access to a SPARQL endpoint for service requests.

Descriptions of basic processes and features that any S-WCMS can use are contained in a SOCCOMAS source code ontology (SC-Basic). Descriptions of special features and peculiarities of a particular S-WCMS are contained in its corresponding instance source code ontology (INST-SCO), which has been individually customized for this particular instance of a SOCCOMAS S-WCMS. The middleware dynamically executes the programming code based on these descriptions. Comparable to a system of building blocks, we use the set of commands and variables known to the middleware to describe and specify all relevant features of an S-WCMS, thereby creating declarative specifications of the S-WCMS that the middleware interprets and dynamically executes.

Based on the declarative specifications contained in SC-Basic and INST-SCO, the specification runs directly. By describing the S-WCMS in a source code ontology one implicitly writes the programming code of the S-WCMS. We call this approach ‘semantic programming’ (28, 58). The commands and variables provided by SPrO serve as an ontology-based language used for describing the GUI, data representations (i.e. composition and specification of HTML components of all data documents in an S-WCMS, including their functionality and input restrictions and logic), user interactions, basic programming logic and all workflow processes of an S-WCMS. No programming in addition to making the descriptions is required as soon as a sufficient set of commands and variables is defined in SPrO, its accompanying middleware and the frontend. This approach, however, requires the development of a middleware (i.e. application tier) that is capable of interpreting all given commands and variables of SPrO and compiling the information of SC-Basic and INST-SCO into an S-WCMS with an automatically generated GUI.

To our knowledge, something like SOCCOMAS has not been realized before. SOCCOMAS applies Semantic Web technology for managing a tuple store, has SPARQL access built into its system rather than having it added as an afterthought and stores data and metadata of an S-WCMS in form of semantic knowledge graphs in its tuple store, making data and metadata accessible for machines through its SPARQL endpoint.

There are other S-WCMS-like frameworks. Apache Stanbol (59), for example, provides components that can be used for extending a content management system with a number of semantic services such as extracting information from contents and documenting them in RDF, thereby semantically enriching them, or providing semantic indexing and search functions. Semantic MediaWiki (60) is an extension of MediaWiki that helps to search, organize, tag, browse, evaluate and share the wiki’s content by adding semantic annotations. Semantic MediaWikis thus represent S-WCMS. Callimachus (61) is a linked data management system for developing semantically enabled web applications, with a strong focus on structured data similar to SOCCOMAS. However, in none of these examples the application itself is controlled by an ontology or SPARQL access is built directly into their system.

Since we entered unknown territory with the semantic programming approach, we could not rely on existing code and concepts and had to develop the corresponding basic engine of SOCCOMAS from scratch. Currently, we are in a development stage that demonstrates, as a proof of concept, that we turned our initial idea into a functional application.

### Basic concept and infrastructure of SOCCOMAS

SOCCOMAS is a ready-to-use application for developing and controlling an S-WCMS. It comprises (see Figure 2) the following:

1) Tuple store framework:
  a) The SPrO [available from (62)] that provides the commands, subcommands, and variables that can be used as a programming language for specifying an S-WCMS (28)
  b) The SC-Basic [available from (63)] that contains a set of basic descriptions that specify various features, workflows and database processes typically required by an S-WCMS
  c) We have developed a set of INST-SCOs for a semantic version of Morph·D·Base, i.e. a morphological S-WCMS that is currently in development (64). Its source code ontologies (MDB-SCOs; available from (65)) contain data views and entry forms specific to semantic Morph·D·Base.
  d) A tuple store framework that stores not only SPrO, SC-Basic and any particular INST-SCO but also all data and metadata statements produced by the users of the S-WCMS. These statements are stored in the form of semantic knowledge graphs. SOCCOMAS uses the Jena tuple store framework, which can be organized into several independent physical RDF stores, with each such store representing a separate ‘workspace’. The workspaces are used to organize the data of the S-WCMS.
2) Middleware and application layer:
  a) A Java-based middleware (available from (66)) that interprets the descriptions from SC-Basic and any INST-SCO and executes all user-triggered interactions
  b) A NoSQL MongoDB that is used for efficient session handling
3) Frontend:
  a) An HTML5/CSS3-based frontend (available from (67)) with a GUI that is specified by the middleware based on the descriptions from SC-Basic and any INST-SCO
  b) A controlled SPARQL endpoint for searching and accessing published data in the knowledge base of the S-WCMS

It is important to note that SOCCOMAS is not restricted to a particular data scheme or knowledge domain. It is developed to allow the set-up of scientific S-WCMSs that are individually customizable. Customization includes the specification of workflows and database processes and different types of data documents with their accompanying entry forms, all of which do not require a specific informatics background except for experience with ontology editors in order to be able to create the descriptions using terms from SPrO. The overall look of the GUI is easily customizable by altering and expanding the well-documented HTML and CSS code of the frontend.

All data of an S-WCMS are uploaded, edited, organized, published and accessed through data documents. Each particular data document possesses its own persistent URI (The URI is generated following the pattern ‘namespace/resource/UniqueNumber-YearMonthDay-DocumentType-VersionNumber’), which can be used to get either an RDF version using the SPARQL endpoint of the S-WCMS or an HTML version through a web browser. In the future, this will be handled through content negotiation.

Currently, each S-WCMS run by SOCCOMAS possesses a very basic access rights system that allows collaborative editing of unpublished data documents [We plan to improve the access rights system to differentiate various access rights categories (read-only, edit, publish etc.) that can be assigned to individual users as well as to defined groups of users.]. When a data document is published and thus becomes openly accessible in the web, it receives its own digital object identifier and is no longer editable, thus enabling persistence of citations.

**Figure 3:**
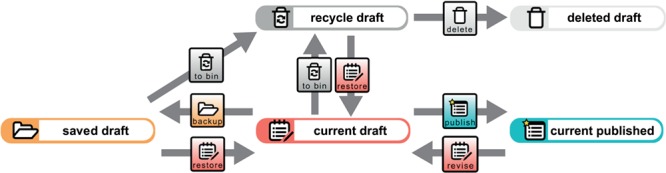
Data document life cycle. The creation of a new data document results in a current draft version of that data document. A copy of the current draft version can be created and subsequently accessed as a saved draft version by triggering the `backup’ process for it. Several different saved draft versions can exist at a time but only one current draft version of a given data document. The current draft version will be moved to the recycle bin and be subsequently accessible as a recycle draft version by triggering the ‘to bin’ process for it. This will result in a lack of a current draft version. However, one of the saved draft or recycle draft versions can be selected to become the new current draft version by triggering the ‘restore’ process for it. Triggering this process is only possible if no current draft version exists. A saved draft version can also be moved to the recycle bin by triggering the ‘to bin’ process for it. A recycle draft version can be completely deleted by triggering the ‘delete’ process for it. All data will be deleted except for the metadata referring to the ‘delete’ process, which can be accessed subsequently through the deleted draft version. However, if this has been the last remaining recycle bin version of this document and no other saved or current draft and no current or previously published version exists anymore for this data document, the entire data record will be completely deleted. A current draft version can be published and thus moved from the draft workspace to the published workspace by triggering the ‘publish’ process. This results in a new current published version of the data document and the deletion of all saved, recycled and deleted draft versions. If a current published version already exists, it will become a previously published version. All previously published versions point to the new current published version. A new revision can be started from the current published version by triggering the ‘revise’ process. This will move a copy of the current published version to the draft workspace, which subsequently can be accessed as a new current draft version. This, however, is only possible if no draft version of this data document. All transition steps triggered for a given data document are tracked in the document’s change history knowledge graph (see Figure 5).

**Figure 4:**
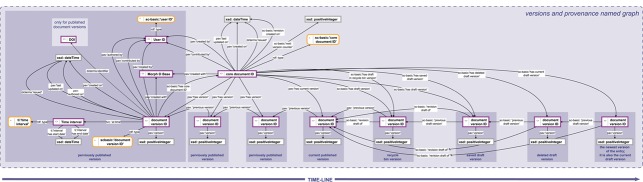
Provenance and versioning knowledge graph. The provenance and versioning knowledge graph for a SOCCOMAS data document produced with semantic Morph·D·Base. The graph is stored in the ‘versions and provenance named graph’ belonging to a particular SOCCOMAS data document. The triple statements indicating the provenance of the different versions of the document are only shown for the oldest (i.e. leftmost) document version ID. dcterms, Dublin Core Terms (75); pav, Provenance, Authoring and Versioning ontology (69, 70); rdf, Resource Description Framework (10); sc-basic, SOCCOMAS source code ontology; ti, Time Interval ontology (73); tvc, the Time-indexed Value in Context ontology (72); xsd: XML Schema (74). For reasons of clarity, resources are not represented with their URIs but with labels. Yellow-bordered box, ontology class; purple-bordered box, instance; gray-bordered box, value or label; dashed-bordered box, named graph.

### Main engine of SOCCOMAS

SPrO together with its accompanying Java-based middleware establishes a semantic language for describing an S-WCMS. The interaction between SPrO, SC-Basic, INST-SCO and the middleware is essential to SOCCOMAS and establishes its main engine.

The commands and subcommands are defined in SPrO as annotation properties, whereas values and variable-carrying resources are ontology individuals (i.e. instances of ontology classes) (28). Relations between resources can be described using specific SPrO object properties. SPrO data properties are used for specifying numerical values or literals for resources that describe the S-WCMS. The descriptions themselves are added, depending on whether they describe general features of any S-WCMS or features that are specific to a particular S-WCMS, to SC-Basic or to the particular INST-SCO, respectively.

The descriptions take the form of annotations of ontology classes and ontology individuals. Each annotation consists of a command, followed by a value, index or resource, and can be extended by axiom annotations that contain subcommands, values and variables taken from SPrO. In the case of ontology individuals, the annotations can also be extended by property assertions.

**Figure 5:**
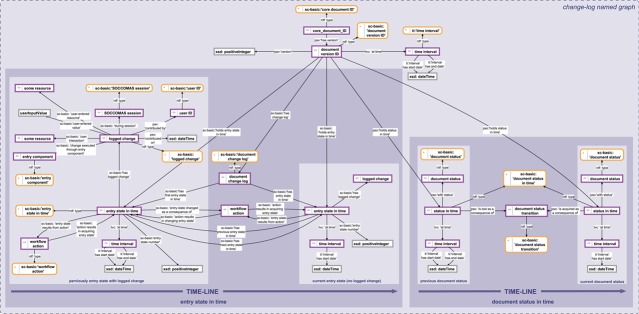
Change history knowledge graph. The change history knowledge graph for a SOCCOMAS data document. The graph is stored in the ‘change-log named graph’ belonging to a particular SOCCOMAS data document. The logged change tracks the entry state in time (left) and the document status in time (right). The logged change documents the date and time of an individual editing step together with the user ID, the session ID, the new resource or value that the user specified and the particular input field that the user used. This logged change is linked to the entry state that the editing step changes. The editing step results in a new entry state in time with a yet empty logged change. Several editing steps result in a chain of entry states in time. The document status in time documents the current status of the document (this refers to the data document life cycle, see Figure 3). It is linked to the previous status through the particular status transition process that led to the current status. In this way, all previous statuses are linked to each other through the chain of particular status transition processes triggered by users. pav, Provenance, Authoring and Versioning ontology (69, 70); pso, Publishing Status Ontology (71, 72); rdf: Resource Description Framework (10); sc-basic, SOCCOMAS source code ontology; ti, Time Interval ontology (73); tvc, the Time-indexe Value in Context ontology (72); xsd, XML Schema (74). For reasons of clarity, resources are not represented with their URIs but with labels. Yellow-bordered box, ontology class; purple-bordered box, instance; gray-bordered box, value orlabel; dashed-bordered box, named graph.

The descriptions in SC-Basic and any particular INST-SCO cover various features of a scientific S-WCMS and can be modified, extended and adapted to the individual needs of any organization or any specific project. SC-Basic describes user administration, covering the description of signup and login forms, user registration and login processes, as well as session management and the description of the template form of a user document. It also describes the general organization and structure of the underlying Jena tuple store framework (68) into six different workspaces, each of which is a separate physical RDF store within the tuple store framework. SC-Basic’s descriptions cover all life cycle processes of a data document (Figure 3). Last but not least, SC-Basic describes automatic tracking procedures, in which (i) user contributions to any given data document are tracked for both the document and the user, (ii) the overall provenance (Figure 4) of a data document is tracked and (iii) a detailed change history (Figure 5) is being logged for each editing step a user conducts for any given document version. All the information gathered through these tracking procedures is recorded in RDF following established data and metadata standards using terms and their corresponding URIs from established ontologies.

**Figure 6:**
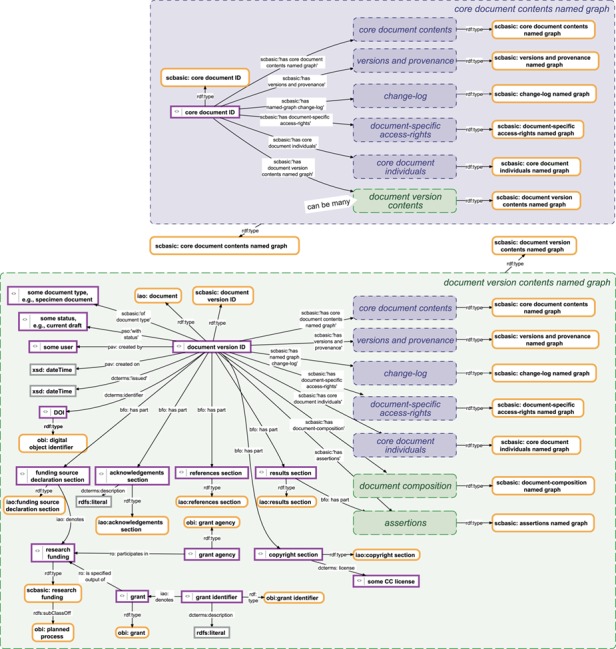
Data management knowledge graphs for a document and its versions. Top: The data management knowledge graph for a SOCCOMAS data document. The graph is stored in the ‘core document contents named graph’ belonging to a particular SOCCOMAS data document. The graph lists the core document ID and links it to various named graphs that contain different kinds of metadata, including the ‘versions and provenance’ named graph (see Figure 4), the ‘change-log’ named graph (see Figure 5), the ‘document-specific access-rights’ named graph (contains access-rights specifications), the ‘core document individuals’ named graph (lists all instances generated for this document together with their rdf:type specification), the ‘document version contents’ named graphs of all versions of the document and the ‘core document contents’ named graph itself. Bottom: The data management knowledge graph for a particular version of a SOCCOMAS data document. The graph is stored in the ‘document version contents named graph’ belonging to the particular version. The graph lists the document version ID and links it to various named graphs that contain different kinds of data and metadata, including the ‘core document contents’ named graph of the document, the ‘versions and provenance’ named graph (see Figure 4), the ‘change-log’ named graph (see Figure 5), the ‘document-specific access-rights’ named graph (contains access-rights specifications), the ‘core document individuals’ named graph (lists all instances generated for this document together with their rdf:type specification), the ‘document composition’ named graphs (specifies the entry form of the version) and the ‘assertions’ named graph (contains the actual assertions, i.e. data). Additional named graphs may be listed depending on the type of data document. Further information is specified relating that is required for efficient searching and version-specific metadata. pav, Provenance, Authoring and Versioning ontology (69, 70); pso, Publishing Status Ontology (71, 72); rdf, Resource Description Framework (10); sc-basic, SOCCOMAS source code ontology; ti, Time Interval ontology (73); tvc, The Time-indexed Value in Context ontology (72); xsd, XML Schema (74). For reasons of clarity, resources are not represented with their URIs but with labels. Yellow-bordered box, ontology class; purple-bordered box, instance; gray-bordered box, value or label; blue narrow-dashed-bordered box, named graph stored in the core workspace; green broad-dashed-bordered box, named graph stored in the draft or published workspace.

A particular INST-SCO, on the other hand, describes all HTML templates for entry forms used in the particular S-WCMS, including all the entry forms and data views for the various types of data documents of this S-WCMS. INST-SCO furthermore covers the specification of the input control and overall behavior of each input field, including the underlying data scheme that specifies how user input triggers the generation of data-scheme-compliant triple statements and where these triple statements must be saved in the Jena tuple store in terms of named graphs and workspaces. A named graph identifies a set of triple statements by adding the URI of the named graph to each triple belonging to this named graph, thus turning the triple into a quad. The Jena tuple store can handle such quadruples. The use of named graphs enables partitioning data in an RDF store and enables making statements about statements comparable to OWL reification, but outperforms the latter for more complex queries (76).

The underlying Jena tuple store framework is organized into six different workspaces. The ‘ontology workspace’ stores SPrO, SC-Basic and the particular INST-SCO for the respective S-WCMS. The ‘admin workspace’ stores user-specific data and metadata. The ‘core workspace’ stores all version-independent data and metadata of a given data document, whereas the ‘draft workspace’ and the ‘published workspace’ store all version-dependent data and metadata of non-published and published versions of a given data document, respectively. The ‘external ontologies workspace’ stores all classes of external ontologies (e.g. from domain reference ontologies used for documenting the data) only with the corresponding label and definition triples. It is connected with a Lucene index directory (77) to handle efficient text-based SPARQL queries. Each workspace is further structured into various different named graphs, with each named graph having its own URI. These named graphs are modeled as ontology instances. Each named graph instantiates a specific ontology class. This allows SOCCOMAS to differentially store the data belonging to a specific document or a specific version of a document into different named graphs. The network of different named graphs belonging to a data document is being set up during the creation life cycle process of a data document. The set of named graphs belonging to a given document can be found in the document’s ‘core document contents named graph’. This named graph also lists the ‘document version contents named graph’ of each particular version that exists of the document. The ‘document version contents named graph’, in turn, lists all named graphs belonging to a particular document version (Figure 6).

The structuring of a workspace into various instances of named graphs of different classes not only facilitatesdata retrieval and data safety but also allows flexible and meaningful fragmentation of data [for a discussion see (36, 49)]. Moreover, the use of named graphs allows SOCCOMAS to store data and metadata associated with a particular version of a document in two ways: (i) in specific named graphs that contain the data and metadata according to the specified data scheme for a machine-readable version and (ii) in the current structure of the particular entry form of this version with all user input for a HTML representation of the data and metadata and thus for a human-readable version. If the version is a draft version, corresponding named graphs are located in the draft workspace; if it is a published version, they are located in the published workspace. Whereas the published workspace can be made publicly accessible via an open SPARQL endpoint, the draft workspace is only accessibly for logged in users with sufficient access rights. All version-independent metadata associated with a given data document are located in corresponding named graphs in the core workspace (Figure 6).

The Java-based middleware interprets the descriptions from SC-Basic and any particular INST-SCO and produces the respective S-WCMS and coordinates its overall operation based on the information from the descriptions contained in SC-Basic and INST-SCO. This includes not only interpreting the descriptions of entry forms and the overall architecture of the GUI and communicating these interpretations with the frontend but also interpreting the data of the particular S-WCMS in reference to the information contained in SC-Basic and INST-SCO. It also includes interpreting the user input communicated from the frontend and processing it in accordance with the corresponding descriptions from SC-Basic and INST-SCO. In other words, the middleware mediates between SC-Basic, INST-SCO and all semantic knowledge graphs in the underlying Jena tuple store framework on the one hand and the browser-based GUI with the user interaction on the other hand (Figure 2). For loading, copying, saving and searching data and metadata in the underlying tuple store framework, the middleware utilizes particular Java classes and methods, which are called through specific trigger points, i.e. URIs of specific SPrO annotation properties, used in description within SC-Basic and INST-SCO and the specific organization of its tuple store framework in terms of different workspaces and named graphs.

### List of functions of SOCCOMAS

Each S-WCMS powered by SOCCOMAS provides a comprehensive, yet very flexible data management tool that meets the growing and constantly changing requirements of organizations and research institutes for storing and processing FAIR data and metadata. It supports the following ontology-driven functionalities:

1) Data control:
  a) intuitive GUI for data retrieval and data input of an S-WCMS, with auto-completion input fields for ontology terms, search and filtering and semantic annotation of free texts;
  b) version-control and document life cycle that allows the saving of work-in-progress copies, controlled publishing and versioning of several publications of the same data document (Figure 3);
  c) provenance transparency provided by the provenance and versioning knowledge graph (Figure 4), with (i) every contributor being tracked, (ii) the possibility to distinguish between the creator of the data document and creator of the data themselves (specification of data-creators is not implemented yet, but will be in future releases of SOCCOMAS) and (iii) the documentation of relevant literature and other metadata;
  d) editing transparency, with every change and every editing step being logged and time-stamped, resulting in a searchable change history knowledge graph (Figure 5) for each data document;
  e) shared semantics over data across all data documents of the same type;
  f) ontology-driven GUI allows changing the description of entry forms within SC-Basic or any particular INST-SCO and the interface will adapt instantly;
  g) SPARQL endpoint allows detailed searches over the various workspaces of an S-WCMS;
2) Data modeling:
  h) by changing the descriptions in SC-Basic or INST-SCO one can
    - re-define the architecture of the GUI components that compose a page in the S-WCMS;
    - re-define the appearance and functionality of each GUI component;
    - change the input control and error-handling of each input field of a given form;
    - re-define the RDF/OWL-compliant data scheme for storing user input (i.e. data);
    - change the location where to store data and therewith change the overall organization of the data;
    - add new data document types with their own specific entry forms and underlying data schemes.

All S-WCMSs based on SOCCOMAS support scientific communication following the FAIR Guiding Principles (29) and provide computer-parsable data and metadata following eScience-compliant standards. Moreover, their published data are Linked Open Data-compliant and enable persistence of citations for all data documents that users publish with the S-WCMS. SOCCOMAS and all S-WCMSs run by it will be entirely built on Semantic Web technologies, thereby providing a maximum of semantic transparency that not only covers data and metadata documentation but also includes the documentation of the application itself. Each S-WCMS run by SOCCOMAS is self-describing.

**Figure 7:**
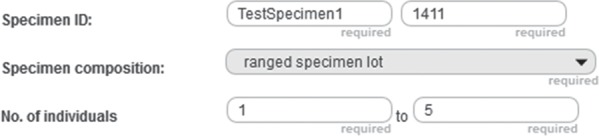
Positive integer input field example. Detail from the entry form of a specimen document from the prototype of semantic Morph·D·Base that is currently in development (https://proto.morphdbase.de/). The positive integer input field discussed in the text is at the bottom right. Here, it contains the value ‘5’ as its input. At its left is the corresponding minimum input field, with the value ‘1’ as its input.

### Example description

In the following, we give an example of how commands and subcommands from SPrO can be used for describing functions and execution procedures of a particular input field of an S-WCMS run by SOCCOMAS. The description is contained in the INST-SCO of that S-WCMS. As an example, we use a positive integer input field of the entry form template of specimen documents described for the semantic version of Morph·D·Base that is currently in development (https://proto.morphdbase.de/). (Semantic Morph·D·Base is a prototype and is fully implemented using semantic programming; even the log in and sign up forms are based on SPrO. If you want to test the prototype, you must first sign up.) The input field is used for specifying the maximum number of individuals of a ranged specimen lot, with a corresponding minimum input field to its left (Figure 7). This description is contained in the corresponding instance source code ontology for specimen documents of semantic Morph·D·Base [SC-MDB-S; available from (65)].

#### Description of the HTML representation of the input field

SPrO can be used for describing the overall composition of an HTML page of a data document (28). Each page is described as a set of HTML elements, which we call entry components. Each entry component is represented in the respective source code ontology as an ontology instance that instantiates a particular ontology class. The composition of entry components of a given page is thus represented as a set of ontology instances and their affiliated ontology classes.

The entry components are linked to each other through the SPrO object property ‘entry component of’ and its inverse property ‘has entry component’, resulting in a nested hierarchy of entry components. A given entry component can have several child entry components, which at their turn also can have several child components, etc. The children of an entry component are represented in HTML as being contained in their parent component. For instance, a label with an input field, another label and another input field being contained in an HTML Document Division Element (<div>) (Figure 7, bottom row). We use the SPrO data property ‘has position in entry component’ to specify for each entry component, in which order, from left to right, it must be represented within its parent component.

**Figure 8:**
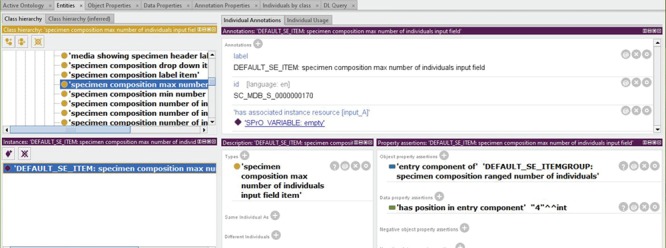
Example of the information associated with a particular entry component. The example is taken from the instance source code ontology SC-MDB-S of the semantic version of Morph·D·Base currently in development (https://proto.morphdbase.de/). Top left: Detail from the class hierarchy of SC-MDB-S, with a focus on the entry component class for a specific maximum integer input field used in an entry form for specimen documents in semantic Morph·D·Base.Bottom left: An ontology instance that represents a particular entry component that instantiates the ontology class currently in focus. This entry component is part of the template form that has been described for specimen documents in semantic Morph·D·Base. The blue background indicates that it is currently selected. Top right: Descriptions belonging to the currently selected ontology instance. The label and ID of the entry component and the SPrO command ‘has associated instance resource [input_A]’, which defines the SPrO variable ‘SPrO_VARIABLE: associated instance resource [input_A]’ initially to be set to ‘SPrO_VARIABLE: empty’. Bottom right: Specification of the parent component of the entry component and its position within this parent compared to its direct sibling entry components. Information visualized using Protégé (http://protege.stanford.edu) (78).

The entire composition of an entry form in form of a nested hierarchy of entry components is represented as an instance-based semantic graph, which is described in the respective source code ontology, stored in its own named graph. The graph functions as a template for the entry form of a particular type of data document. In our example, the type of data document is a specimen document of semantic Morph·D·Base and the template graph is stored within the SC-MDB-S source code ontology. The corresponding description that is linked to the entry component of our example of a positive integer input field is depicted in Figure 8. When a user creates a new specimen document in semantic Morph·D·Base, SOCCOMAS copies the template graph and stores the copy in a particular named graph belonging to the newly created document (document composition named graph, Figure 6). SOCCOMAS interprets this copied graph and produces the HTML representation of this newly created specimen document whenever a browser requests accession to this document.

**Figure 9:**
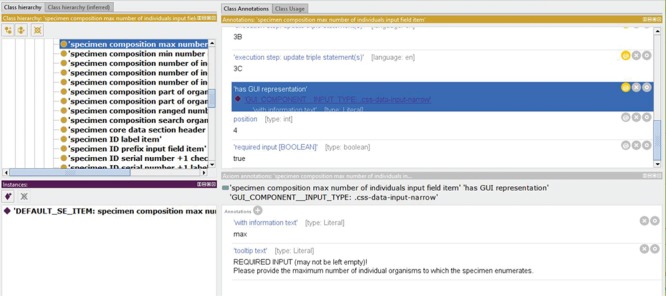
Example of the information associated with an ontology class that is instantiated by a particular entry component. The example is taken from the instance source code ontology SC-MDB-S of the semantic version of Morph·D·Base currently in development (https://proto.morphdbase.de/). Top left: Detail from the class hierarchy of SC-MDB-S, with the entry component class currently selected that is for a specific maximum integer input field used in an entry form for specimen documents in semantic Morph·D·Base. Bottom left: An ontology instance that represents a particular entry component that instantiates the ontology class currently in focus. Contrary to Figure 3, here the ontology instance is not selected. Right top: Excerpt from the descriptions of the ontology class currently selected. The annotation ‘required input [BOOLEAN]’ = true indicates that this input field must be completed for the specimen document to be publishable. The annotation ‘has GUI representation’ is currently in focus. It links to the SPrO value ‘GUI_COMPONENT__INPUT_TYPE: .css-data-input-narrow’, which specifies which CSS-class must be used for visualizing all entry component instances of this ontology class in the GUI. The functionality of the input field, including its input control and interactions with other entry components of the entry form, are described in a sequence of ordered execution steps, of which only execution step 3C ‘update triple statement(s)’ is partially shown. Right bottom: The axiom annotations of the annotation in focus above (i.e. ‘has GUI representation’. The SPrO annotation property ‘tooltip text’ with a literal as its value specifies the information that appears when hovering the mouse over the input field. The SPrO annotation property ‘with information text’ with its literal as value specifies the text that appears in the input field when no input has been provided. Information visualized using Protégé (http://protege.stanford.edu) (79).

The actual HTML representation of a particular entry component is specified in the ontology class it instantiates or in the instance itself. The value linked to the SPrO annotation property ‘has GUI representation’ can be mapped to a particular Cascading Styles Sheets (CSS) class. This CSS class, in turn, specifies how the entry component must be represented in HTML. In our example, the value ‘GUI_COMPONENT__INPUT_TYPE: .css-data-input-narrow’ maps to the CSS class ‘.css-data-input-narrow’, which defines a specific type of input field element such as the one in Figure 7, at the bottom right. Additional axiom annotations are used to specify a tooltip text to appear when hovering with a mouse over the input field, and an information text that appears in the input field as long as no input has been provided by the user (for the respective class descriptions, see Figure 9). The value specified with the SPrO annotation property ‘has associated instance resource [input_A]’ tracks the input from the corresponding minimum input field. Initially, it has no value associated, indicated by the SPrO variable ‘SPrO_VARIABLE: empty’, but it will be updated if a value is entered in the minimum input field.

List of GUI-related RDF-specifications for the input field (including the two axiom annotations):

**Table:** 

entry component instance	spro:‘entry component of’	parent entry component instance	
entry component instance	spro:‘has position in entry component’	‘4’	
entry component instance	spro:‘has associated instance resource [input_A]’	spro:‘SPrO_VARIABLE: empty’	
entry component class	spro:‘has GUI representation’	scbasic:‘GUI_COMPONENT__INPUT_TYPE: css-data-input-narrow’	
		spro:‘with information text’	‘max’
		spro:‘tooltip text’	‘REQUIRED INPUT (may not be left empty)! Please provide the maximum number of individual organisms to which the specimen enumerates.’

#### Descriptionof the input control and functionality of the input field

The HTML representation of each entry component (i.e. its CSS class reference), its input control and its specific functionality, including the data scheme for how user input must be translated into a corresponding semantic graph and where this graph must be stored within the underlying tuple store in terms of named graph and workspace, are described in the ontology class that the entry component instantiates using annotation properties and variables from SPrO (Figure 9).

Some entry components of the entry form of a data document refer to required input. This is also the case with our positive integer input field example. The SPrO annotation property ‘required input [BOOLEAN]’ with the value ‘true’ indicates that input is mandatory for the positive integer input field. SOCCOMAS checks whether the user provided adequate input and in case they have not, SOCCOMAS will notify them when they attempt to publish the document.

##### Execution step 0: trigger

**Figure 10:**
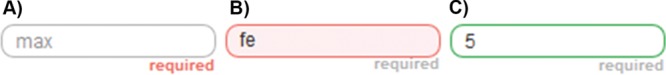
Example of the visual response of the input controls of an application powered by SOCCOMAS. (**A**) The positive integer input field with no user input. ‘required’ is written in red because the input field represents a mandatory input field and it still has no input. ‘max’ is written in light gray inside of the input field, indicating that it should be used to provide the maximum value of a range of two values that must be provided, with the minimum input field taking the position left of this input field (see Figure 2). (**B**) The same input field with a non-positive integer input. The field has a pink background, its border is red, and a temporary error message appears (here not shown) informing the user to use only natural numbers in case the input is not a positive integer or a higher value than the one used in the minimum input field as input. (**C**) The same input field with a positive integer input. The border of the field is green, indicating that the input successfully passed the application’s input control.

The entry component class also contains a sequentially ordered list of execution steps that is triggered through user input. The first execution step in the sequence is described using the SPrO annotation property ‘execution step trigger’. The axiom annotation using the SPrO annotation property ‘has GUI input type’ together with the SPrO value ‘INPUT_CONTROL: positive integer’ defines the input control (Currently, SOCCOMAS provides defined input control for ‘Boolean’, ‘click’, ‘phone number’, ‘email address’, ‘composite literal’, ‘GeoJSON string’, ‘literal’, ‘date’, ‘date time’, ‘decimal degree’, ‘float’, ‘float pH’, ‘float percentage’, ‘geo-coordinate decimal latitude’, ‘geo-coordinate decimal longitude’, ‘integer percentage’ and ‘positive integer’, each with a corresponding error message. Moreover, it provides input control for ontology classes and instances via auto complete of their labels.). It specifies that only positive integer is allowed as input. Additional axiom annotations specify that the input defines a particular SPrO variable (‘SPrO_VARIABLE: user/GUI input [input_B]’ and that only users with the right to edit the document will be able to trigger this sequence of execution steps. SOCCOMAS will check whether the input is a positive integer and indicate success with a green border around the input field and a red border in case of failure (Figure 10). After having successfully passed the input control, SOCCOMAS will continue with execution step 1A.

List of RDF-specifications for execution step 0:

**Table:** 

entry component class	spro: ‘execution step trigger’	‘0’	
		spro:‘has GUI input type’	spro:‘INPUT_CONTROL: positive integer’
		spro:‘input value/ resource defines SPrO variable resource’	spro:‘SPrO_VARIABLE: user/GUI input [input_B]’
		spro:‘requirement for triggering the execution step’	spro:‘SPrO_VARIABLE: edit document-access-right for this document’

##### Execution step 1A: search triple store.

Execution step 1A searches for a specific resource that has been linked to this particular max input field entry component through a specific triple statement. This resource refers to the URI of the particular specimen that the document references. This URI is used when storing the input according to an underlying data scheme. The SPrO annotation properties ‘subject’, ‘property’ and ‘object’ specify the searched triple, with the SPrO variable ‘SPrO_VARIABLE:?’ indicating a placeholder and the SPrO annotation properties ‘load from/save to/update in named graph (this document’s specific individual of)’ and ‘named graph belongs to workspace’ the named graph and workspace where the triple can be found. The SPrO annotation properties ‘search target’ and ‘search target defines SPrO variable’ specify which resource of the triple is the target of the search and writes it to a specific SPrO variable for referencing it in subsequent execution steps.

List of RDF-specifications for execution step 1A:

**Table:** 

entry component class	spro:‘execution step: search triple store’	‘1A’	
		spro:‘subject’	spro:‘SPrO_VARIABLE: this entry component’
		spro:‘property’	spro:‘has associated instance resource [input M]’
		spro:‘object’	spro:‘SPrO_VARIABLE:?’
		spro:‘load from/save to/update in named graph (this document’s specific individual of)’	scbasic:‘document-composition named graph’
		spro:‘named graph belongs to workspace’	scbasic:‘WORKSPACE_ BASIC: draft workspace’
		spro:‘search target’	spro:‘SPrO_VARIABLE: object’
		spro:‘search target defines SPrO variable’	spro:‘SPrO_VARIABLE: associated instance resource [input_M]’

##### Execution step 2A: search triple store

Execution step 2A searches for another value that has been linked to this entry component. This value is the input value of the corresponding minimum input field, which is empty in case no minimum value has been provided yet.

List of RDF-specifications for execution step 2A:

**Table:** 

entry component class	spro:‘execution step: search triple store’	‘2A’	
		spro:‘subject’	spro:‘SPrO_VARIABLE: this entry component’
		spro:‘property’	spro:‘has associated instance resource [input A]’
		spro:‘object’	spro:‘SPrO_VARIABLE:?’
		spro:‘load from/save to/update in named graph (this document’s specific individual of)’	scbasic:‘document- composition named graph’
		spro:‘named graph belongs to workspace’	scbasic:‘WORKSPACE_BASIC: draft workspace’
		spro:‘search target’	spro:‘SPrO_VARIABLE: object’
		spro:‘search target defines SPrO variable’	spro:‘SPrO_VARIABLE: associated instance resource [input_A]’

##### Execution step 2B: if–then–else statement

Execution step 2B executes an if–then–else condition, in which the ‘if’ input value is set to the input that triggered this execution process (i.e. the maximum number of individuals of the specimen lot) and the ‘if’ target value is set to the value identified in step 2A, which is the input of the minimum input field (i.e. the minimum number of individuals of the specimen lot). The ‘if’ operation compares the input value with the target value and returns ‘true’ if the input value is larger than the target value. If this is not the case, it returns ‘false’. If the application returns ‘true’, the process will ‘then’ proceed with execution step 3A, ‘else’ with 2C.

As a side note, it should be mentioned that SOCCOMAS can also handle several if-then-else conditions, but each must be specified as separate execution step and they will be executed in the specified sequence order of steps.

List of RDF-specifications for execution step 2B:

**Table:** 

entry component class	spro:‘execution step: if-then-else statement’	‘2B’	
		spro:‘has IF input value’	spro:‘SPrO_VARIABLE: user/GUI input [input_B]’
		spro:‘has IF target value’	spro:‘SPrO_VARIABLE: associated instance resource [input_A]’
		spro:‘has IF operation’	spro:‘SPrO_IF_OPERATION: SOME input larger than target’
		spro:‘then:’	‘3A’

##### Execution step 2C: decision dialog

Execution step 2C returns an error message to the GUI, informing the user that the value must be higher than the value of the minimum number input field and terminates the process. Nothing will be saved to the tuple store.

List of RDF-specifications for execution step 2C:

**Table:** 

entry component class	spro:‘execution step: decision dialogue’	‘2C’	
		spro:‘application error message’	‘The value must be higher than the value of the minimum number.’
		spro:‘end action operation’	spro:‘SPrO_OPERATION: ERROR end action’

##### Execution step 3A: update triple statement(s)

Execution step 3A updates the input stored for this particular entry component. This guarantees that the input will be shown in the document’s entry form after reloading the HTML page of the specimen document.

List of RDF-specifications for execution step 3A:

**Table:** 

entry component class	spro:‘execution step: update triple statement(s)’	‘3A’	
		spro:‘subject (this document’s specific individual of)’	this entry component class
		spro:‘property’	scbasic:‘has user/GUI input [value_B]’
		spro:‘object’	spro:‘SPrO_VARIABLE: to be updated’
		spro:‘load from/save to/update in named graph (this document’s specific individual of)’	scbasic:‘document- composition named graph’
		spro:‘named graph belongs to workspace’	scbasic:‘WORKSPACE_ BASIC: draft workspace’
		spro:‘update with resource/value’	spro:‘SPrO_VARIABLE: user/GUI input [input_B]’

##### Execution step 3B: update triple statement(s)

Execution step 3B updates the object position of a defined triple statement consisting of the particular resource identified in execution step 1A as the ‘subject’, the data property ‘maximum number of individuals’ as the ‘property’ and the ‘object’ to be updated with the user input that triggered this execution process. The triple is located in the assertions named graph belonging to the particular specimen document being edited and resides in the draft workspace. The semantic knowledge graph located in the assertions named graph is the data graph belonging to this particular specimen document.

List of RDF-specifications for execution step 3B:

**Table:** 

entry component class	spro:‘execution step: update triple statement(s)’	‘3B’	
		spro:‘subject’	spro:‘SPrO_VARIABLE: associated instance resource [input_M]’
		spro:‘property’	scMDBs:‘max number of individuals’
		spro:‘object’	spro:‘SPrO_VARIABLE: to be updated’
		spro:‘load from/save to/update in named graph (this document’s specific individual of)’	scbasic:‘assertions named graph’
		spro:‘named graph belongs to workspace’	scbasic:‘WORKSPACE_ BASIC: draft workspace’
		spro:‘update with resource/value’	spro:‘SPrO_VARIABLE: user/GUI input [input_B]’

##### Execution step 3C: update triple statement(s).

Execution step 3C updates the object position of a triple statement that is linked to the entry component of the minimum input field. It is updated to the value of the maximum input field, which is provided by the user input that triggered this execution process. This value is needed for when user input is provided for the minimum input field, in which case the minimum number input must be compared to this maximum number input with the requirement to be smaller than the maximum input value.

List of RDF-specifications for execution step 3C:

**Table:** 

entry component class	spro:‘execution step: update triple statement(s)’	‘3C’	
		spro:‘subject (this document’s specific individual of)’	minimum number of individuals input field entry component class
		spro:‘property’	spro:‘has associated instance resource [input_B]’
		spro:‘object’	spro:‘SPrO_VARIABLE: to be updated’
		spro:‘load from/save to/update in named graph (this document’s specific individual of)’	scbasic:‘document-composition named graph’
		spro:‘named graph belongs to workspace’	scbasic:‘WORKSPACE_ BASIC: draft workspace’
		spro:‘update with resource/value’	spro:‘SPrO_VARIABLE: user/GUI input [input_B]’

##### Execution step 3D: trigger workflow action

Execution step 3D triggers the ‘edit’ SOCCOMAS workflow action, which in turn triggers the automatic tracking procedures that track the user’s contribution to this document, update the document’s provenance metadata and log the edit action in the document’s change history.

List of RDF-specifications for execution step 3D:

**Table:** 

entry component class	spro:‘execution step: trigger workflow action’	‘3D’	
		spro:‘triggers workflow action’	scbasic:‘SOCCOMAS_ BASIC_WORKFLOW_ ACTION: edit’

## Synergetic effects and advantages of SOCCOMAS

### Centralized development but still flexible customization

Basic functions and features are implemented by SOCCOMAS and shared by all S-WCMSs driven by SOCCOMAS. This enables easy set-up of an S-WCMS, even for small projects that usually have no funding for developing their own S-WCMS. With SOCCOMAS and within the limits set by the commands and variables provided by SPrO and its accompanying Java middleware, domain experts can customize their S-WCMS to suit their own institutional needs. All customization is modular and may involve not only the specification of a particular look for the GUI (by specifying their own CSS file), but also the specification of particular entry forms, data document types and underlying data schemes as well as particular workflows through customizing the INST-SCO. Moreover, any third party can develop additional functions (e.g. implementation of analytic tools for refining the basic functionality and interface of a particular S-WCMS) and make the corresponding new commands and variables with the accompanying middleware functionality openly available through additional source code ontologies and accompanying Java classes and methods.

SOCCOMAS’s underlying modular architecture and the separation between SPrO, SC-Basic and any particular INST-SCO will enable the introduction of new commands and variables or new widgets that may be required for developing new functions and adapting to new standards. They can be implemented in all existing S-WCMS by updating and complementing the various ontologies and accompanying Java classes or methods used by SOCCOMAS.

Moreover, since the specifications for any S-WCMS run by SOCCOMAS are contained in its corresponding INST-SCO, it can be used as a template for specifying the source code ontology for a similar S-WCMS, only having to adjust the descriptions to the requirements specific to this particular S-WCMS. For semantic Morph·D·Base, for example, we use several different source code ontologies. We have a basic source code ontology (SC-MDB-Basic) that contains descriptions for the features and processes that all the different data modules within semantic Morph·D·Base share, and we have a separate source code ontology for each data module within semantic Morph·D·Base, including one for specimen documents (SC-MDB-S) and one for morphological description documents (SC-MDB-MD). Now, if somebody wants to develop their own S-WCMS for specimen data, they could use not only SPrO and SC-Basic but also SC-MDB-Basic together with SC-MDB-S and adjust the latter two to meet the requirements of their particular S-WCMS for specimen data, thereby saving valuable development resources.

In case somebody wants to change some of the defined SOCCOMAS processes such as the document status transitions belonging to the document life cycle or somebody wants to change the data scheme underlying, for instance, the change history tracking procedure, they can change respective descriptions in SC-Basic.

### Comparability and Linked Open Data–enabled

All S-WCMSs based on SOCCOMAS will be fully comparable in terms of cross-visualizing their data and metadata views. They all share the same data schemes for basic metadata such as provenance, change-log, access rights, and versioning, which is especially interesting in the context of interdisciplinary research. In the future, SOCCOMAS will also enable the convenient inter-linking of different data documents within a particular S-WCMS. Such inter-linking possibilities can be readily extended to allow inter-linking of data documents across all the different S-WCMSs run by SOCCOMAS. Moreover, import- and export-functions of data and metadata can be easily specified across particular S-WCMSs, because they are run by the same engine and based on the same SPrO, which SOCCOMAS’ middleware can interpret.

Each S-WCMS that is based on SOCCOMAS is also Linked Open Data–compliant and FAIR, as its contents are documented in semantic knowledge graphs, the contents of which are (i) accessible and referable via stable URLs, (ii) are linked to vocabularies and ontologies that express their meaning, (iii) can be accessed and searched through the S-WCMS’s SPARQL endpoint, with which data and metadata can be exported in a variety of formats (RDF/XML, RDF/JSON, JSON-LD, Turtle, N-Triples), and (iv) are amendable to open licenses as soon as they are published (i.e. moved from the draft to the published workspace by triggering the ‘publish’ document status transaction). When appropriable, an S-WCMS will leverage available Linked Open Data to serve tools like text annotation, geo-coding, and citation management. Users of an S-WCMS that is based on SOCCOMAS are guided to interlink their data with existing resources on the Web or resources from the S-WCMS itself.

### Flexible GUI development

The composition, structure and functionality of the GUI of an S-WCMS based on SOCCOMAS are described in SC-Basic and the corresponding INST-SCO and are thus controlled through SOCCOMAS. As a consequence, changes in the basic composition of the GUI can be conducted on-the-fly, which allows adapting it to user demands at any point. As a consequence, when the middleware has been set up, the GUI of an S-WCMS can be developed through a user-centered design approach, by enabling the domain experts and thus the main users of the S-WCMS to adjust the entry components of any given page without the intervention of software programmers.

## Conclusion and outlook

Although RDF tuple stores handle large data volumes well and facilitate detailed data retrieval and data inferences, they have yet to become the prime database technology for content management systems. One reason may be found in the lack of adequate application development frameworks that are well integrated with RDF, i.e. that allow reading, writing, updating and searching semantic knowledge graphs and visualizing contents from the graph in HTML (17) [for initial attempts see e.g. (18–27)]. It is not enough to be able to store and query data from a tuple store. Application developers need means to provide possibilities for users to interact with the data and metadata graphs through entry forms and various data visualizations.

SOCCOMAS is the first step toward such a development framework. With SOCCOMAS you can develop GUIs that are easy to use for any user, providing conventional HTML entry forms with easy to read and interpret user-input. User input triggers the generation of often rather complicated semantic data and metadata knowledge graphs, which will be stored in the background into various named graphs of the respective S-WCMS. For users who access the data via the web portal of the S-WCMS, the semantic knowledge graphs are hidden behind HTML pages. The graphs can, however, be consumed by various applications through the SPARQL endpoint of the S-WCMS and are thus available for querying and analyses. As a consequence, all S-WCMSs based on SOCCOMAS support scientific communication following the FAIR Guiding Principles (29) (see also discussion above, 3.1) and provide computer-parsable data and metadata following eScience-compliant standards. And with the HTML display of contents from the underlying semantic knowledge graph, SOCCOMAS bridges the gap between the Semantic Web of data and the classical web of applications and pages.

Our use case of developing a specimen document module for semantic Morph·D·Base has proven that using SOCCOMAS and semantic programming saves valuable resources and development time. The source code ontology (SC-MDB-S) for this module has been written by a domain expert with knowledge in ontology engineering but no expertise in any programming language. Development time was substantially reduced by the fact that many processes and functionalities for this module have been automatically provided by SOCCOMAS so that development was restricted only to features specific to this particular module. Another reason for the reduced development time is the fact that SOCCOMAS allows for one-layer development of an S-WCMS—as opposed to the common three-layer development (i.e. back end, middleware, front end) for other data base systems—because all its specifications are made in source code ontologies. The use case has also shown that changes to the overall organization of the GUI or the addition of new input fields can be conducted on-the-fly by only having to change the respective descriptions in SC-MDB-S, which facilitates a user-centered design approach to application development.

At the moment, developing and editing an S-WCMS based on SOCCOMAS still requires, in addition to a minimum of technical programming background, experience with an ontology editor such as Protégé. We plan to develop an editor with GUI for SOCCOMAS itself. This editor should be usable by domain experts (i.e. experts on some subject matter) as a one-stop application for developing a particular S-WCMS. Domain experts with no informatics background should be able to use this editor to set up their own S-WCMS for collaboration with other experts through the Internet.

SOCCOMAS stands for full semantic transparency: each S-WCMS powered by SOCCOMAS allows the documentation of how data and metadata are produced and what they mean, of the data’s provenance and editing history as well as who contributed to it and how each data document is interlinked with other documents in the S-WCMS. Since SOCCOMAS and its S-WCMSs are based on semantic programming and described in source code ontologies, the SOCCOMAS application itself is semantically transparent—SOCCOMAS is self-describing.

Our next steps will be to test the scalability of SOCCOMAS and compare storage space required for similar data documents between SOCCOMAS and relational database applications. We plan to migrate the data from the relational Morph·D·Base to the semantic Morph·D·Base, which will allow us to compare the two systems. We will also test how well specific information is findable and accessible through the SPARQL endpoint of the semantic Morph·D·Base as compared to the same information in the relational Morph·D·Base.

## Availability of Data and Materials

SPrO is available for free under the GNU Lesser General Public License Version 3 (LGPL) at https://github.com/SemanticProgramming/SPrO.

The accompanying Java-based interpreter and the interface are both available for free under the LGPL 3 at https://github.com/SemanticProgramming/Interpreter and https://github.com/SemanticProgramming/Interface, respectively.

SC-Basic is available for free under the LGPL 3 at https://github.com/SemanticProgramming/SOCCOMAS.

SC-MDB-Basic, SC-MDB-S and SC-MDB-MD are available for free under the LGPL 3 at https://github.com/SemanticProgramming/SemMorphDBase.

The prototype of Semantic Morph·D·Base serves as a proof of concept of the applicability of SPrO and semantic programming and can be found at (https://proto.morphdbase.de/).

## Notes

**Database URL:** https://github.com/SemanticProgramming/SOCCOMAS
